# Ruptured Metastatic Pheochromocytoma: A Nightmare to the Surgeon

**DOI:** 10.7759/cureus.36890

**Published:** 2023-03-29

**Authors:** Shridhar Hosamani, Deepak Ghuliani, Rajshekar Puttaswamy

**Affiliations:** 1 Department of Surgery, Maulana Azad Medical College, New Delhi, IND; 2 Department of Surgical Oncology, Dr. Ram Manohar Lohia Institute of Medical Sciences, Lucknow, IND

**Keywords:** spontaneous rupture of tumor, adrenal tumor, shock, acute abdomen, pheochromacytoma

## Abstract

Pheochromocytoma is a neuroendocrine tumor arising from the adrenal medulla, which often causes an adrenalin rush in the treating surgeon and the anesthesiologist. The tumor rupture presenting as acute abdomen and shock is a rare and life-threatening event. We present a case report of ruptured metastatic pheochromocytoma in a 37-year-old patient whose signs and symptoms included acute abdomen and shock. The exact mechanism causing the spontaneous rupture of a tumor is not clearly understood although some theories are proposed. CT scan is the most important investigation in this emergency. A high index of suspicion is needed to diagnose rupture in a known case of pheochromocytoma presenting with acute abdomen and shock. Ruptured pheochromocytoma is a rare emergency, and timely and precise diagnosis and initial primary nonsurgical approach followed by elective surgical resection are to be practiced whenever possible. When emergency exploration is imminent, a multi-specialty team should be prepared with a well-equipped ICU, rapid hemodynamic monitoring and corrections, massive blood transfusion protocol, and timely resuscitation.

## Introduction

Pheochromocytoma is a neuroendocrine tumor arising from the adrenal medulla, which not only causes a catecholamine rush in patients but also an adrenaline rush in the treating surgeon and the anesthesiologist. It is a rare tumor that can lead to a disastrous outcome, if unrecognized. The symptoms are mostly due to catecholamine release, and they mimic a plethora of conditions including hypoglycemia, hyperthyroidism, coronary artery disease, heart failure, stroke, and panic disorder, to name a few [1]. The tumor rupture presenting as acute abdomen and shock is a rare and life-threatening event. We present a case of ruptured metastatic pheochromocytoma presenting in shock with acute abdomen.

## Case presentation

A 37-year-old female presented to the surgical emergency with sudden onset of severe abdominal pain, non-colicky in nature, associated with one episode of vomiting followed by the collapse of the patient. She denied having had any recent abdominal trauma or any similar complaints in the past. Several months prior to this episode, she was diagnosed as hypertensive and was on multiple antihypertension drugs. In addition, she also had complaints of abnormally excessive sweating and palpitation episodes.

At presentation, she was pale and drowsy with a pulse rate of 130 bpm, low volume, respiratory rate of 26 per min, and systolic blood pressure of 66 mmHg with cold peripheries. On examination, her abdomen was tense and distended with diffuse tenderness. A provisional diagnosis of ruptured abdominal viscera with hypovolemic shock was made.

She was resuscitated with crystalloids, blood, and blood products. On evaluation, she was found to have hemoglobin 6.1 g/dL, hematocrit of 18.9%, total leukocyte count of 9900 cells/mm^3^, and platelet 269,000/mL with normal coagulation profile.

**Figure 1:**
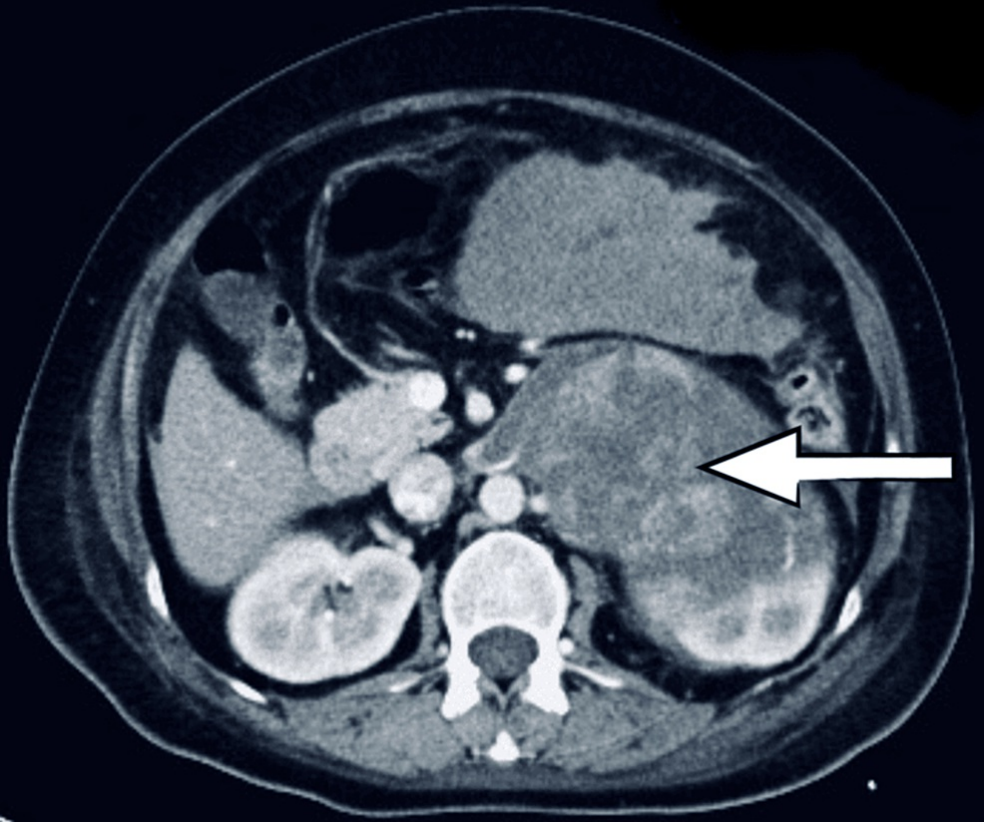
Contrast-enhanced CT abdomen (axial section) showing a large heterogeneously enhancing left adrenal mass with ?rupture with hemoperitoneum (solid white arrow).

After hemodynamic stabilization, plain abdomen X-ray was done, which showed no evidence of any pneumoperitoneum; USG abdomen revealed moderate free fluid with internal echoes. Contrast-enhanced CT (CECT) of the abdomen showed a large heterogeneously enhancing left adrenal mass with rupture into the lesser sac with hemoperitoneum (Figure 1) and multiple lytic lesions in lumbar vertebrae L2-L5 were noted. The 24-hour urinary metanephrine test came out to be within normal range. Emergent transarterial embolization (TAE) was attempted, which failed due to no single bleeding vessel amenable to embolization being visualized.

In view of her hemodynamic instability, CECT findings, and failed TAE, the patient was taken up for exploratory laparotomy. On entry into the abdomen cavity, a pool of blood was encountered in the left upper abdomen; the tumor had multiple feeding vessels with profuse bleeding and was densely adhered to surrounding structures. In view of dense adhesions with the kidney and diffuse bleeding from the tumor, left adrenalectomy along with a left nephrectomy were done.

Postoperatively, the patient was shifted to the intensive care unit for continued resuscitation and close vitals monitoring. However, the patient continued to have labile blood pressure, continued to deteriorate, and later developed arrhythmia and succumbed.

**Figure 2:**
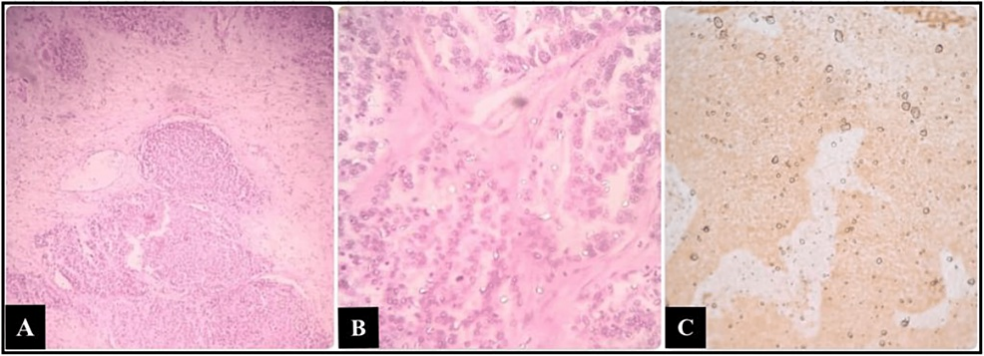
Microphotographs showing: (A, B) Hemotoxylin & eosin staining of the left adrenal mass showing zellballen (small nests or alveolar pattern), trabecular or solid patterns of polygonal/spindle-shaped cells in rich vascular network; (C) Immunohistochemical staining of the left adrenal mass specimen showing positive synaptophysin stain.

Histopathological examination of the specimen was conclusive of adrenal pheochromocytoma with areas of hemorrhage and necrosis with pheochromocytoma of the adrenal gland scaled score (PASS) of 13 (Figure 2).

## Discussion

Since the first account of pheochromocytoma was published by Felix Frankel in 1886 [2], the tumor continues to deceive clinicians, even though the diagnostic and management strategies have evolved drastically. Spectrums of symptoms are described for pheochromocytoma but the headache, episodic sweating, and palpitations constitute the classical presentation [1]. Though hypertension is the most common finding found in pheochromocytoma patients, it is estimated that it is responsible for only 0.5% of all hypertensives, on account of its rarity. Classically pheochromocytoma is called 10% tumor as it is 10% familial, 10% bilateral, 10% seen in a pediatric population, 10% extra-adrenal, and 10% malignant [1]. The WHO’s 2017 classification classified extra-adrenal pheochromocytoma as paraganglioma, based on anatomic location as histologically both are identical. Also the term ‘malignant pheochromocytoma’ is replaced by ‘metastatic pheochromocytoma’ as the presence of metastases is the only proof of its malignant nature [3]. Although a number of histologic scoring systems have come up to predict the malignant potential, no single criteria has been appreciated to date.

Rupture of pheochromocytoma is the rarest complication of this tumor resulting in intra-abdominal hemorrhage and shock. Only a handful of cases are reported. The bleeding can be isolated to the retroperitoneum only or may involve the peritoneal cavity also. The exact mechanism causing the spontaneous rupture of the tumor is not clearly understood and some theories are proposed.

The high pressure on the tumor capsule is caused by rapid tumor growth or intratumoral bleeds leading to the tearing of the capsule. The drastic fluctuation in blood pressure resulting in an abrupt decrease in blood flow to the rapidly growing tumor causes tumor necrosis and as the blood flow is restored, the rushing blood leads to hemorrhage from these necrotic areas. A similar mechanism of avascular necrosis and tumor bleeding is proposed in alpha-adrenergic blockade [4]. Although no case of rupture of metastatic pheochromocytoma has been reported so far, the relationship between the invasive nature of metastatic pheochromocytoma, and the size of the tumor with tumor rupture is not known.

A high index of suspicion is needed to diagnose rupture in a known case of pheochromocytoma presenting with acute abdomen and shock. In a previously unknown case presenting, for the first time, with acute abdomen and shock, the precise diagnosis of ruptured pheochromocytoma is extremely difficult leading to inadvertent exploration and catastrophe. The CT scan is the most important investigation in this emergency. Along with adrenal mass, it will also show the bleeding inside and around the tumor, retroperitoneal and intraperitoneal bleeding, hematoma, and sometimes, rent in the capsule of the tumor. Most importantly, it rules out other causes of acute abdomen. Biochemical evaluation with either plasma metanephrines or 24-hour urinary metanephrines is the workhorse in confirming the diagnosis [5].

Historically, resection of the adrenal gland, along with the tumor, was met with high mortality and yet, was the only available option. As learned over many years, preoperative blood pressure optimization by combined alpha and beta-adrenergic blockade to prevent an intraoperative hypertensive crisis is the cornerstone in managing these patients. Implementing this in an emergent situation is challenging and is not possible most of the time. Initial conservative management with fluid resuscitation, blood and blood products transfusion, and transarterial embolization of the adrenal artery followed by elective resection after optimization of blood pressure is advocated. If this protocol is followed, the mortality rate is similar to elective resection of pheochromocytoma [4]. In a review of the literature, Hanna et al. identified 50 documented cases between 1944 and 2010, of which 12 involved spontaneous intra-peritoneal hemorrhages. A review of these 12 cases revealed that emergent laparotomy resulted in a mortality of 29% [6].

Massive bleeding, pulmonary edema, cardiac arrhythmias, and heart failure are the main culprits causing death. In some cases, surgical exploration is inevitable and provides the only hope for saving patients. Transarterial embolization of adrenal arteries as a therapeutic option for Pheochromocytoma was first described in 1978 by Brunan and colleagues [7]. At present, it has a limited role in managing pheochromocytoma due to adverse effects like tumor necrosis, but it comes as a handy tool in controlling bleeding in emergency tumor rupture [8]. Availability and restricted success rates are its limitations.

## Conclusions

Ruptured metastatic pheochromocytoma is a rare emergency, and timely and precise diagnosis and initial primary nonsurgical approach followed by elective surgical resection are to be practiced whenever possible. When emergency exploration is imminent, the anesthetists and surgical team should be prepared with their armamentarium of a well-equipped intensive care unit, rapid hemodynamic monitoring and corrections, massive blood transfusion protocol, and timely resuscitation.
